# The effect of subpressure on the bond strength of resin to zirconia ceramic

**DOI:** 10.1371/journal.pone.0179668

**Published:** 2017-06-22

**Authors:** Yong-Mei Li, Rui-Shen Zhuge, Zu-Tai Zhang, Yue-Ming Tian, Ning Ding

**Affiliations:** School of Stomatology, Capital Medical University, Beijing, China; Nanjing Medical University, CHINA

## Abstract

**Objective:**

This study was conducted to investigate the effect of subpressure on the bond strength of resin to zirconia ceramic. The subpressure would create a pressure gradient which could clean out the bubbles in the adhesives or bonding interface.

**Methods:**

Twenty-eight pre-sintered zirconia discs were fabricated. Half of them were polished (group P, n = 14), and the rest were sandblasted (group S, n = 14). After sintered,the surface roughness of the zirconia discs was measured. Then, they were randomly divided into two subgroups (n = 7). The groups were named as follows: PC: P + no additional treatments; PP: P + 0.04 MPa after application of adhesives; SC: S + no additional treatments; and SP: S + 0.04 MPa after application of adhesives. Resin columns were bonded to the zirconia specimens to determine shear bond strength (SBS). The bonding interfaces were observed and the fracture modes were evaluated. Statistical analysis was performed on all data.

**Results:**

The surface roughness of group S was significantly higher than that of group P (P<0.05). The SBS values were PC = 13.48 ± 0.7 MPa, PP = 15.22 ± 0.8 MPa, SC = 17.23 ± 0.7 MPa and SP = 21.68 ± 1.4 MPa. There were significant differences among the groups (P<0.05). Scanning electron microscopy (SEM) results showed that the adhesives of group SP and PP were closer and denser to the zirconia ceramic than that of group PC and SC. The proportion of the mixed fracture mode significantly increased after adding subpressure (P< 0.05).

**Conclusion:**

Subpressure can improve the shear bond strength of resin to zirconia ceramics and increase micro-infiltration between the adhesives and the zirconia ceramics, especially on the rough surfaces.

## 1. Introduction

With the increasing demand for esthetics in prosthodontic treatments, various all-ceramic restorations have been widely used in clinical settings [1]. Zirconia demonstrates superior mechanical properties, chemical stability, biocompatibility and tooth-like color [2] compared with other ceramic materials, making it a commonly used material in dentistry for all-ceramic crowns [3], posts [4], orthodontic brackets [5,6] and even implants [7,8]. However, the conventional cementation techniques do not provide an adequate level of bond strength for the zirconia [9]. This is because etching with hydrofluoric acid and silanization had no positive effects on the zirconia bonding due to its resistance to acids and the absence of silicon oxide [10].

Many surface treatments have been used to improve the bond strength of resin to zirconia ceramics over the past two decades, including air abrasion [11], diamond burs abrasion [12], silica (tribochemical) coating [13], silicoating [14, 15],selective infiltration etching [16] and laser [17, 18]. However, many studies have found microscopic cracks because these surface treatments can accelerate tetragonal-to-monoclinic (t→m) phase transformation [19–22], and other researchers found that airborne particle abrasion decreased micro tensile bond strength [23]. Additionally, the addition of coatings can result in poor adhesion to the zirconia [24]. Previous research has suggested that sandblasting before sintering is a useful method for significantly increasing the surface roughness, and it is considered to reverse the transformation (m→t) caused by sandblasting during the sintering process [19,25]. Additionally, Nobutaka et al. [26] focused on the effect of a primer on bonding to the zirconia. Wei et al. [27] suggested that a high pressure blowing method could improve the adhesion of resin to dentin.

The subpressure infiltration technique is based on the vacuum infiltration theory [28, 29], which is a simple and one-step technique to close micro-voids, repair material defects and develop various kinds of biomaterial scaffolds [30].The purpose of the current study was to evaluate the effect of a novel subpressure infiltration technique on the bonding of resin to zirconia.

## 2. Materials & methods

**Fig 1:**
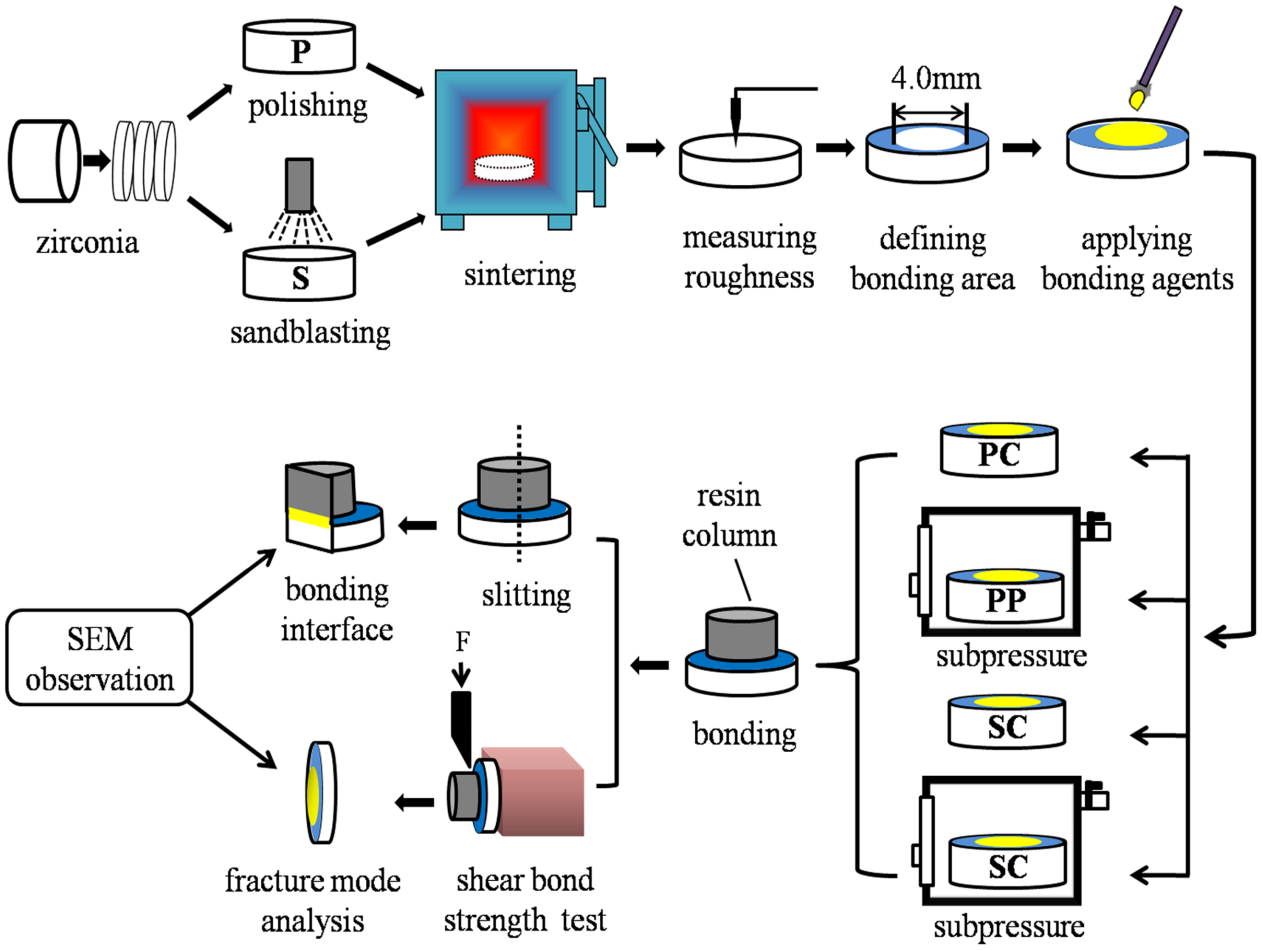
The flow chart of the experiment.

Fig 1 shows the flow chart of the experiment. The process in detail was as follows.

### 2.1 Preparation of the zirconia discs

Twenty-eight discs (16 mm in diameter, 1.5 mm in thickness) were fabricated from pre-sintered yttria-stabilized tetragonal zirconia polycrystal (Y-TZP) ceramic blocks (High transparent material, Nissin-Metec China Co., Ltd., China) with a diamond saw (Isomet 4000 Linear Precision Saw, Buehler, USA) under copious water. Half of the discs were polished (P) using 1200 grit water-proof silicone-carbide paper, the other half were sandblasted (S) (TJK-SP II, Tianjin Haide, China) using 120 μm Al_2_O_3_ particles at 2 bar blasting pressure from a distance of 10 mm for 10 sec. All zirconia discs were densely sintered (Kawo Everest Therm, Germany) at a heating speed of 5°C/min to 1450°C, holding for 2 h, and then naturally cooling to room temperature. All zirconia discs were ultrasonically cleansed in deionized water for 10 min to remove surface debris prior to use,dried with clean air flow and kept in a desiccator.

### 2.2 Surface roughness

The surface roughness of the zirconia ceramic was measured after being densely sintered with a surface roughness measuring instrument (JB-4C, Shanghai Taiming Optical Instrument Co., Ltd., China), and the zirconia ceramic surfaces were observed by 3-D roughness reconstruction with scanning electron microscopy (Phenom-world Co., LTD., Netherlands, SEM).

### 2.3 Subpressure infiltration technique

**Fig 2:**
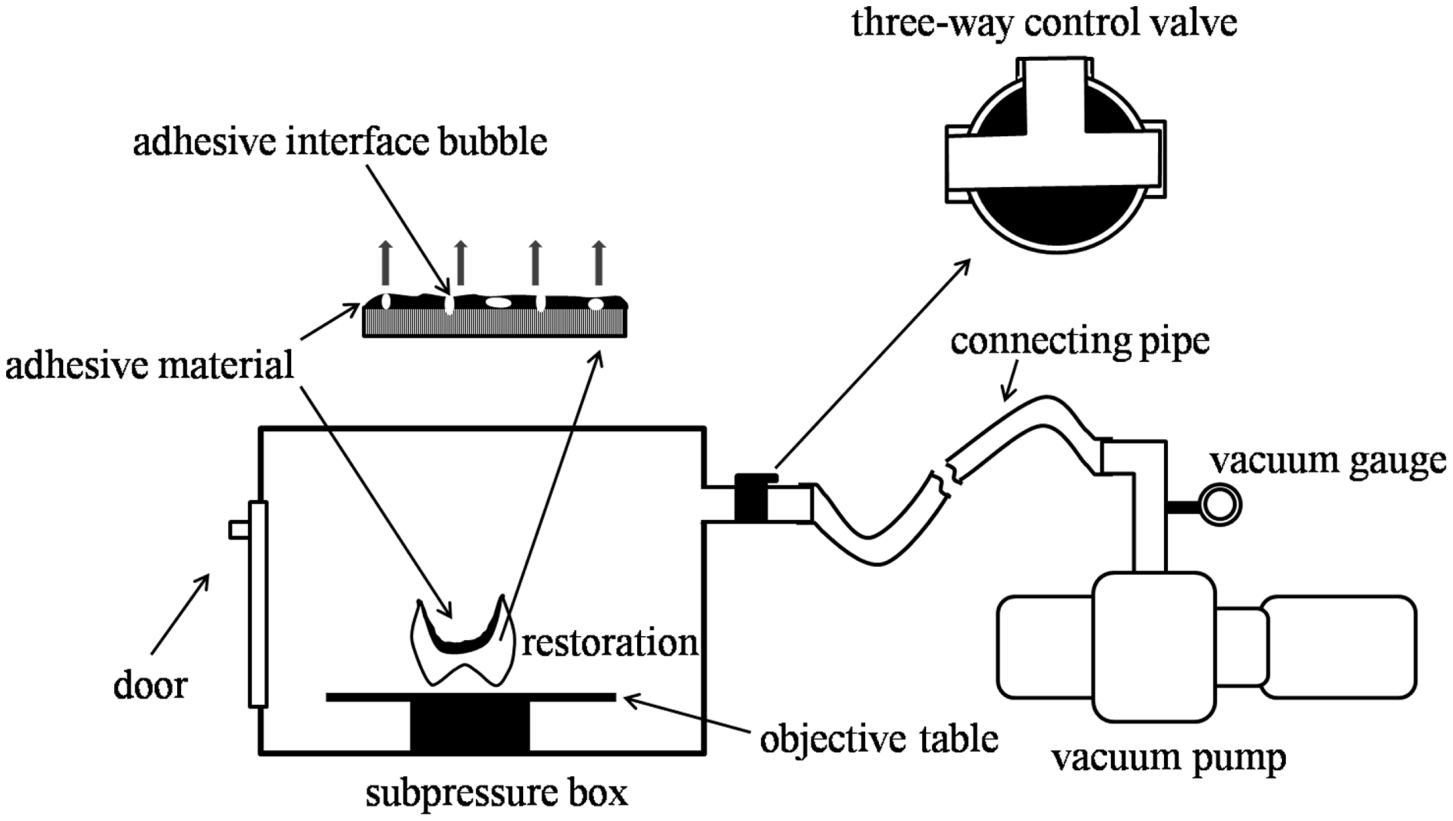
The schematic diagram of the subpressure infiltration technique.

In this study, the novel subpressure device consists of a subpressure box, a vacuum pump, a vacuum gauge, and a three-way valve. The schematic diagram of the subpressure infiltration technique is shown in Fig 2. The specimens were put in the subpressure box through the door. The vacuum gauge can adjust the pressure in the subpressure box, and by turning the three-way valve outside, the inner pressure in the subpressure box could return to atmospheric pressure.

### 2.4 Specimen of resin to zirconia

Prior to the application of subpressure, group P (polished) and S (sandblasted) were randomly divided into two subgroups, respectively, labeled PC: P + no additional treatments, PP: P + 0.04 MPa after applying adhesives, SC: S + no additional treatments, and SP: S + treated with 0.04 MPa after applying adhesives.

A piece of transparent tape (20 μm in thickness) with a circular hole (4 mm in diameter) was placed on the zirconia surface to define the bonding area. The primer and luting agents (Variolink N, Ivoclar Vivadent AG, Liechtenstein) were applied to the zirconia surface according to the manufacturer’s instructions. Group PP and SP were placed in the subpressure box under 0.04 MPa for 3 min after applying the primer and luting agents, respectively (For the mechanism, see preceding **2. 3**). The resin column (5 mm in diameter, 4 mm in thickness) was made of composite resin (Valux^™^ Plus, 3M ESPE, USA) with a stainless steel mold in advance. Then, the resin column was put on the zirconia surface, and covered over the hole of transparent tape exactly. 20 N of force was loaded on the resin column for 1 min before curing from three directions for 10 sec with a light curing unit (Elipar^™^ 2500, 3M ESPE, USA) and holding for 3 min. All specimens were stored in distilled water at 37°C for 24 hours. Finally, zirconia substrates of each specimen were embedded in methyl methacrylate resin.

### 2.5 Bonding interface observation

One specimen was randomly selected from each group and sectioned perpendicular to the bonding surface to observe the bonding interface by SEM.

### 2.6 Shear bond strength test

Shear bond strength (SBS) test was performed using a universal testing machine (AG-X Plus, Shimadzu Co., LTD., Shimadzu, Japan) at a cross-head speed of 0.5 mm/min until the resin column was separated from the zirconia substrate. The SBS values were calculated according to the following formula:

$${\text{SBS = F}}_{\text{max }}\text{/ S}$$

where SBS is shear bond strength (MPa), F_max_ is maximum load (N), and S is the cross-section surface area (mm^2^).

### 2.7 Fractured mode analysis

The debonded surfaces of the specimens were inspected by SEM (500× magnification) to assess the fracture modes, which were classified as the following:

1. Cohesive fracture: A fracture within the resin layer, adhesives or zirconia.
2. Interface fracture: A fracture at the adhesive-zirconia or resin-adhesive interface.
3. Mixed fracture: Both cohesive and interface fractures were observed in the same disc.

### 2.8 Statistical analysis

Student’s t-test was performed to evaluate the differences in roughness between group P and S. Two-way ANOVA was performed to study the contributions of the subpressure, the sandblast and the interaction of these factors on the SBS. A chi-square test was used to examine the difference in the distribution of the fracture modes (SPSS 19.0 for Windows, SPSS Inc., USA). The statistical significance level was set at α = 0.05.

## 3. Results

### 3.1 Surface roughness

**Fig 3:**
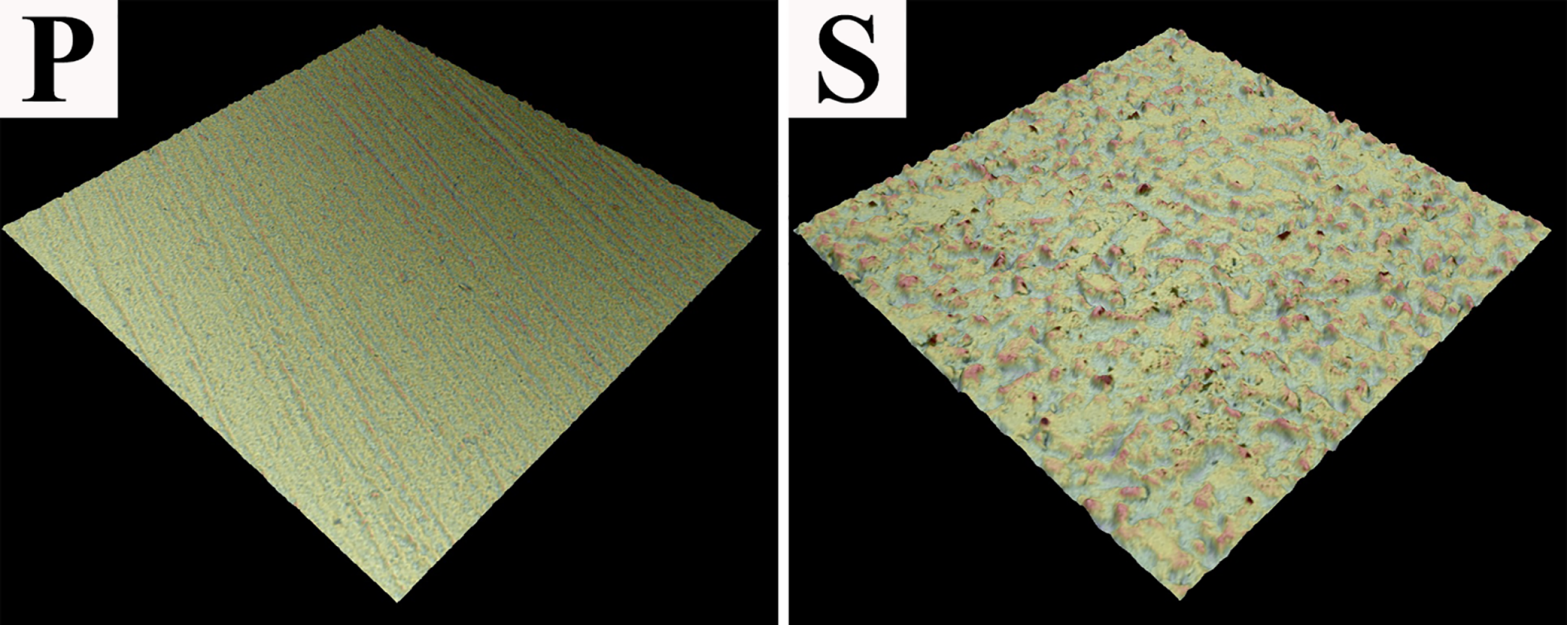
Representative 3-D images of sintered zirconia in group P and S.

The surface roughness values of group P and S were 0.92 ± 0.81 μm and 3.13 ± 1.01 μm, respectively, and there was a significant difference between them (P<0.05). Representative 3-D images are shown in Fig 3. Group S showed more irregular outlines compared with group P in which the surface was more planar.

### 3.2 Bonding interface observation

**Fig 4:**
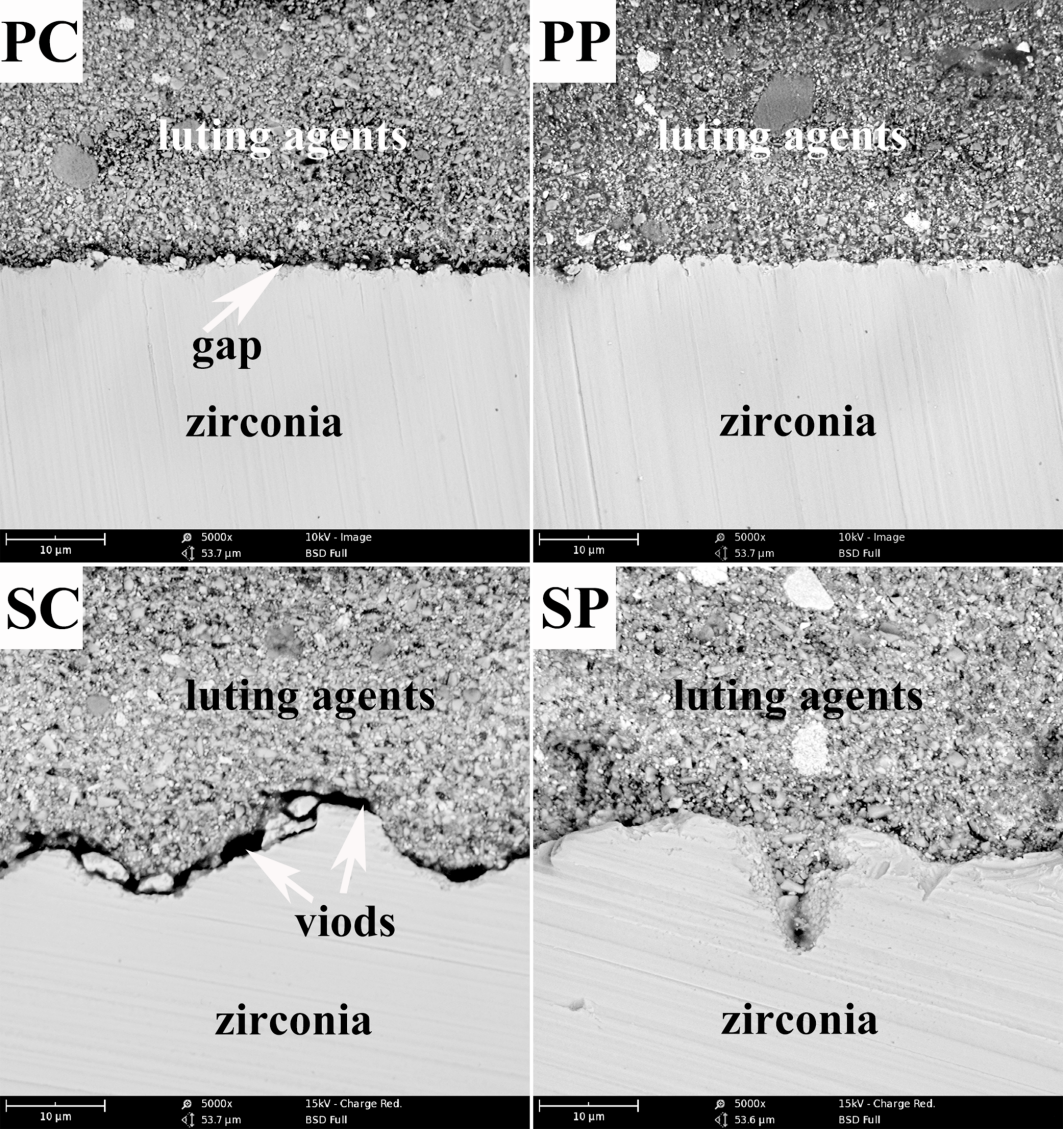
Representative SEM microphotographs of all groups (5000 ×).

Fig 4 shows the bonding interfaces of the resin to the zirconia. Compared with group P, the zirconia surface of group S was wavier and more uneven. There was an obvious gap (arrow) in subgroup PC between the adhesives and the zirconia, whereas the bonding interface was uniform and intact with no voids or defects in subgroup PP. The adhesives were able to be added into the micro fissures of the zirconia surface in subgroup SP, while there were some voids (arrow) between the adhesives and the zirconia in subgroup SC.

### 3.3 Shear bond strength test

**Fig 5:**
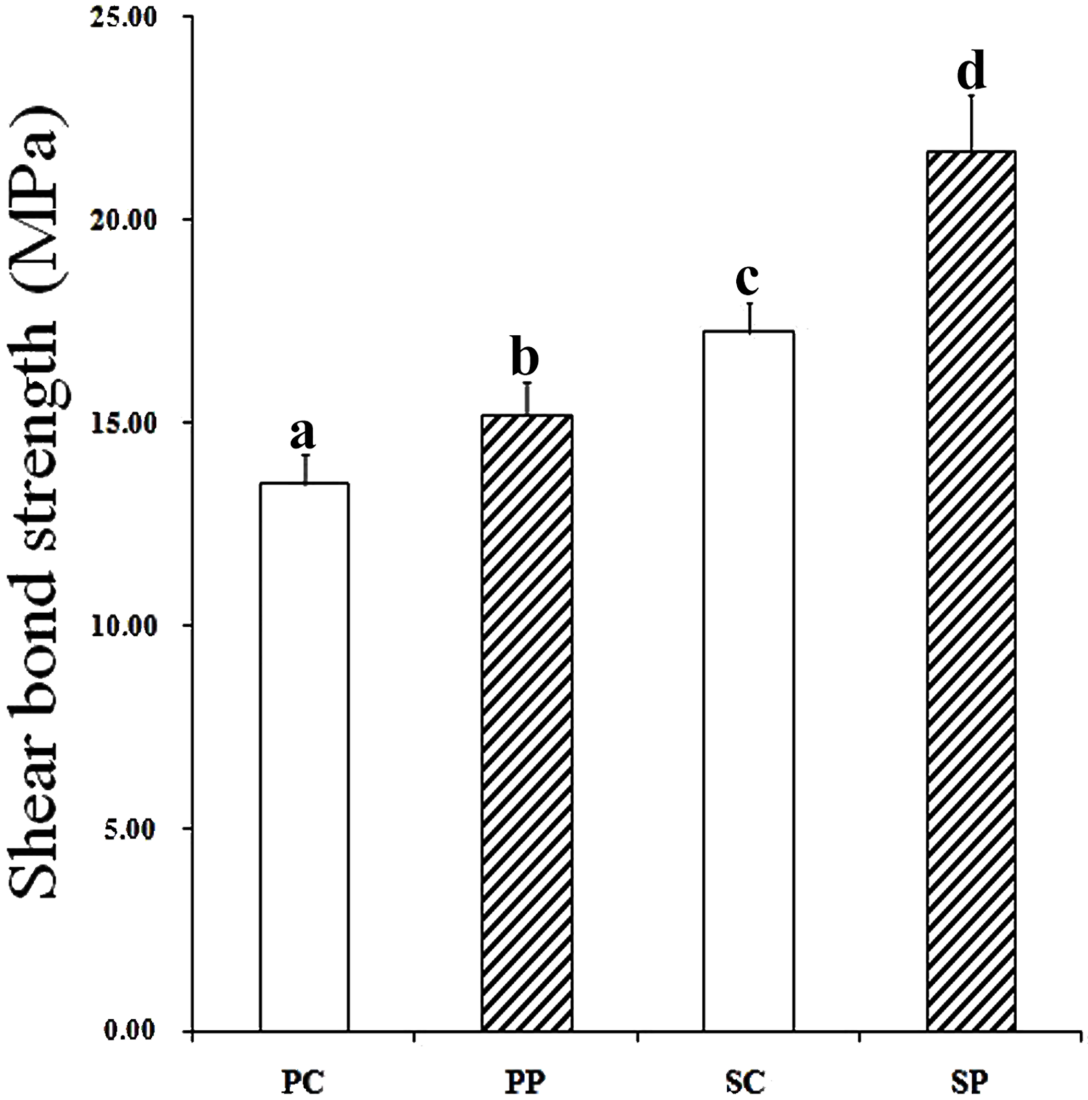
Shear bond strength of all groups. Different letters indicate a significant difference (P < 0.05).

The mean values and standard deviations of the SBS values were PC = 13.48 ± 0.7 MPa, PP = 15.22 ± 0.8 MPa, SC = 17.23 ± 0.7 MPa, and SP = 21.68 ± 1.4 MPa. There was a significant difference in the SBS among the groups (P<0.05) (Fig 5). Two-way ANOVA test showed that both subpressure and sandblast had significant effects to the SBS of resin to zirconia ceramics, and there were significant interaction between them (Table 1).

**Table 1:** Test of Two-way ANOVA.

Source	Type III Sum of Squares	df	Mean Square	F	Sig.
Corrected Model	224.644^a^	3	74.881	83.19	0
Intercept	6855.316	1	6855.316	7615.932	0
subpressure	57.289	1	57.289	63.645	0
sandblast	156.366	1	156.366	173.715	0
subpressure * sandblast	10.989	1	10.989	12.208	0.002
Error	18.003	20	0.9		
Total	7097.962	24			
Corrected Total	242.646	23			

R Squared = .926(Adjusted R Squared = .915)

### 3.4 Fracture mode analysis

Table 2 shows the distribution of the fracture modes after the SBS test. No cohesive fracture was observed in any group. The fracture mode distribution of group PP and SC was the same and was significantly different compared with group SP and PC (P<0.05). Mixed fractures had the highest frequency in group SP, while the group PC showed the lowest percentage of mixed fractures. These groups were significantly different (P<0.05).

**Table 2:** Distribution (%) of fracture modes.

Fracture mode	PC	PP	SC	SP
Cohesive fracture	0	0	0	0
Interface fracture	83.3% (5)	50% (3)	50% (3)	33.3% (2)
Mixed fracture	16.7% (1)	50% (3)	50% (3)	66.7% (4)

The chi-square analysis revealed that there was a significant difference in the fracture mode between groups (P< 0.05) except between group PP and SC (P>0.05).

**Fig 6:**
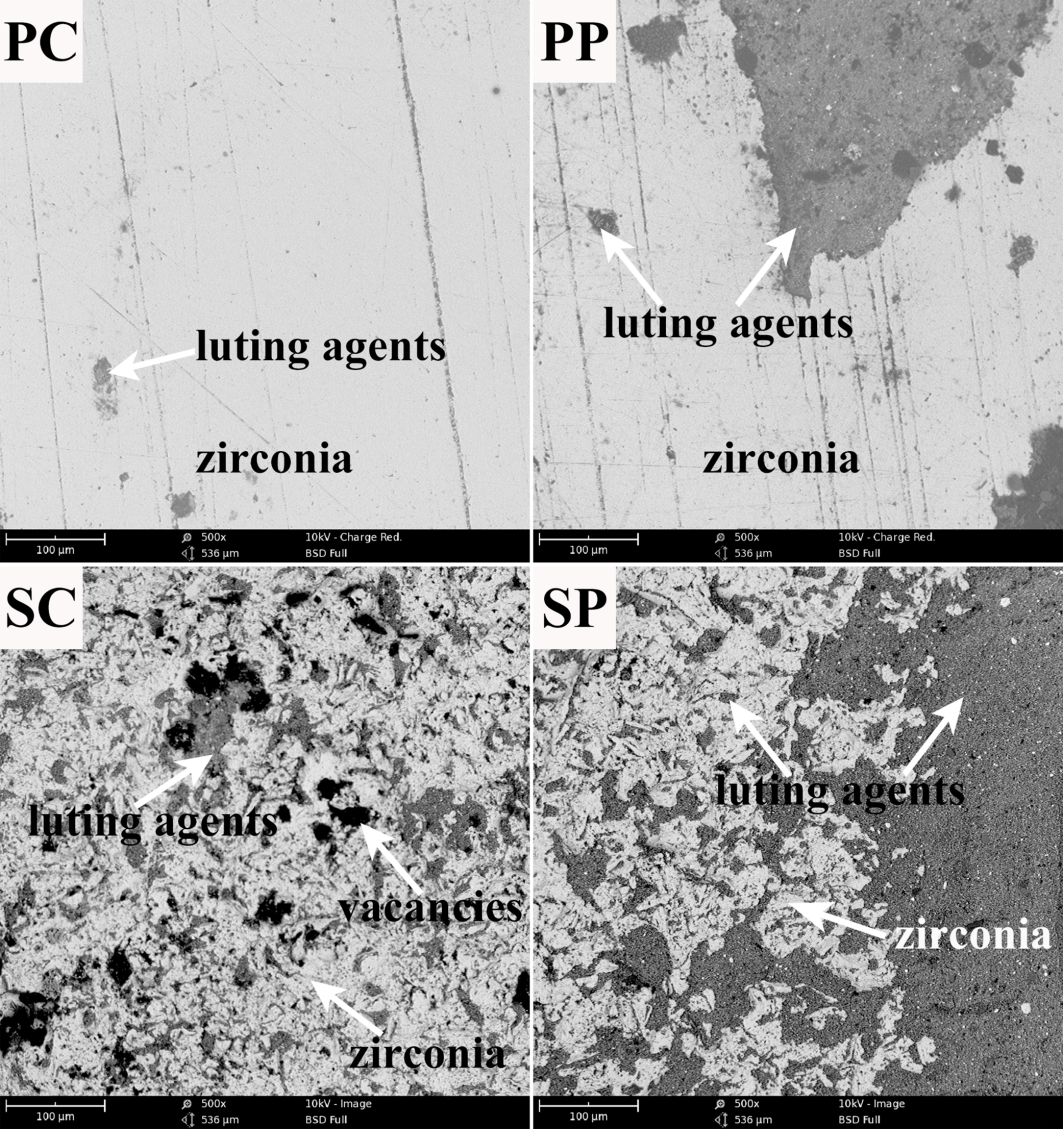
Representative fracture modes of all groups (500 ×).

As Fig 6 shows, there were few adhesives left on the surface of the zirconia in group PC. In group PP, more adhesives could be observed on the zirconia surface. In group SC, there were still some scattered vacancies (arrow) without adhesives filling them. In group SP, most adhesives remained on the zirconia surface even in the micro pits and micro fissures. Additionally, some resin left on the zirconia surface was visible to the naked eye.

## 4. Discussion

At present, there are amounts of cases where the retention of restorations is reliant on bonding. Poor bonding may result in restorations becoming loose or dislodged, or it may produce micro-leakage, resulting in plaque accumulation, which can lead to secondary caries, marginal gingivitis, etc. [31]. Hence, the quality of the bonding is of increasing importance and is a dominant factor required for long-term success, especially in regard to acid resistance and silica-free zirconia ceramic restorations. Although many dental techniques [32] have been extensively investigated to improve the bond strength of resin to zirconia ceramics, there is no established protocol that can produce a stable bond which can be easily used at the present time. To achieve higher retention, prevent micro-leakage, and increase the fracture and fatigue resistance of restorations, many studies are under way [13, 26, 27]. However, the subpressure infiltration technique has not been applied in the dental adhesion.

Previous studies have suggested that roughness affects the bond strength [17, 33, 34]. The purpose of this study was to investigate the effect of subpressure on bonding to different rough surfaces. The surface roughness of group S was significantly higher than that of group P (Fig 3). In addition, the SBS values of group S was significantly higher due to the sandblasting (P<0.05), which was in agreement with the findings of other researchers [19, 33, 34].

In this study, the subpressure infiltration technique was as follows. First, the zirconia surface was covered with adhesives. When the coated zirconia specimens were held in the subpressure chamber, the bubbles between the zirconia and the adhesives or in the adhesives were exhausted due to the pressure gradient. When recovering to atmospheric pressure, the pressure on the adhesives pushed them into the voids or fissures of the zirconia surface, which made the adhesives close to the zirconia substrates.

As the subpressure infiltration technique mechanism mentioned, SEM results (Fig 4) verified that the adhesives were in closer contact with the zirconia in the subpressure groups (SP and PP); whereas space still remained on the surface of the non-subpressure zirconia specimens, especially on the rougher surface. The SBS of the subpressure group was higher than that of the non-subpressure group, confirming that the subpressure had a significant effect on the bonding. The distribution of the mixed fracture mode was significantly different (P < 0.05) under subpressure and sandblasting, which was in agreement with the results of the SBS in this study.

The current theories of mechanics suggest that the adhesive must penetrate into the voids within the adherent surface, and the air bubbles on the interface should be cleared out as possible to promote adhesion. The subpressure could effectively remove air on the interface and amplify the penetration of the adhesives into the micro-pits and micro-hollows of the zirconia surface, which would consequently increase the bonding area and the mechanical locking function [35, 36]. In addition, the close combination of the adhesives to the zirconia increased the intermolecular forces, and the subpressure infiltration technique could potentially reduce microleakage and improve the bonding property [37, 38].

Furthermore, the viscosity of the adhesives materials, the solvent in the primer or the luting agent could affect the infiltration of the zirconia. Low viscosity of the adhesives is conducive and will allow faster infiltration of the gap. Usually, the strength of low-viscosity adhesives is lower than that of high-viscosity adhesives [39, 40]. Additionally, primers or luting agents containing functional monomers could increase the chemical bonding and wettability [26,41]. The configuration of the zirconia surface could affect the infiltration as well, and the subpressure value and action time could also affect the infiltration capacity. Further studies are being conducted.

Finally, compared to other treatments on the zirconia, the subpressure infiltration technique had the advantages of lower technical sensitivity, low cost, short processes and a wide application to other materials. Additionally, the subpressure infiltration technique was not limited to the shape of the restorations.

## 5. Conclusions

This in vitro study suggests that the subpressure is an efficient method of improving the bond strength of resin to zirconia, and it is more effective on a rough surface.

## Supporting information

S1 Text Raw data for SBS. (DOCX)

S1 Fig The physical map of subpressure box. (TIF)
