# Supplementary material for: The effect of subpressure on the bond strength of resin to zirconia ceramic
Source: PLoS One. 2017 Jun 22;12(6):e0179668. doi: 10.1371/journal.pone.0179668 (PMC5480956; doi:10.1371/journal.pone.0179668)
Supplement: S1 Text — (DOCX) [file pone.0179668.s001.docx]

Raw data

| Maximum load: F max (N) | | | | | | |
| --- | --- | --- | --- | --- | --- | --- |
| PC | 165.68 | 178.35 | 160.42 | 159.46 | 170.39 | 181.47 |
| PP | 203.42 | 192.58 | 183.86 | 177.19 | 201.11 | 188.51 |
| SC | 213.83 | 215.97 | 231.32 | 218.26 | 215.39 | 203.69 |
| SP | 251.29 | 265.06 | 279.59 | 260.39 | 276.31 | 300.86 |
| area of bonding: S 12.56 mm^2^ | | | | | | |
| Shear bond strength (SBS) (MPa) | | | | | | |
| PC | 13.19 | 14.20 | 12.77 | 12.70 | 13.57 | 14.45 |
| PP | 16.20 | 15.33 | 14.64 | 14.11 | 16.01 | 15.01 |
| SC | 17.02 | 17.20 | 18.42 | 17.38 | 17.15 | 16.22 |
| SP | 20.01 | 21.10 | 22.26 | 20.73 | 22.00 | 23.95 |

| **Descriptives** | | | | | | | | | | |
| --- | --- | --- | --- | --- | --- | --- | --- | --- | --- | --- |
| SBS | | | | | | | | | | |
|  | | N | Mean | Std. Deviation | Std. Error | 95% Confidence Interval for Mean | | Minimum | Maximum | Between- Component Variance |
|  |  |  |  |  |  | Lower Bound | Upper Bound |  |  |  |
| PC | | 6 | 13.4800 | .72993 | .29799 | 12.7140 | 14.2460 | 12.70 | 14.45 |  |
| PP | | 6 | 15.2167 | .80114 | .32706 | 14.3759 | 16.0574 | 14.11 | 16.20 |  |
| SC | | 6 | 17.2317 | .70853 | .28926 | 16.4881 | 17.9752 | 16.22 | 18.42 |  |
| SP | | 6 | 21.6750 | 1.38704 | .56626 | 20.2194 | 23.1306 | 20.01 | 23.95 |  |
| Total | | 24 | 16.9008 | 3.24805 | .66301 | 15.5293 | 18.2724 | 12.70 | 23.95 |  |
| Model | Fixed Effects |  |  | .94875 | .19366 | 16.4969 | 17.3048 |  |  |  |
|  | Random Effects |  |  |  | 1.76637 | 11.2795 | 22.5222 |  |  | 12.33019 |

| **Test of Homogeneity of Variances** | | | |
| --- | --- | --- | --- |
| SBS | | | |
| Levene Statistic | df1 | df2 | Sig. |
| 1.517 | 3 | 20 | .241 |

| **ANOVA** | | | | | |
| --- | --- | --- | --- | --- | --- |
| SBS | | | | | |
|  | Sum of Squares | df | Mean Square | F | Sig. |
| Between Groups | 224.644 | 3 | 74.881 | 83.190 | .000 |
| Within Groups | 18.003 | 20 | .900 |  |  |
| Total | 242.646 | 23 |  |  |  |

**Post Hoc Tests**

| **Multiple Comparisons** | | | | | | | |
| --- | --- | --- | --- | --- | --- | --- | --- |
| Dependent Variable: SBS | | | | | | | |
|  | (I) Group | (J) Group | Mean Difference (I-J) | Std. Error | Sig. | 95% Confidence Interval | |
|  |  |  |  |  |  | Lower Bound | Upper Bound |
| LSD | PC | PP | -1.73667^*^ | .54776 | .005 | -2.8793 | -.5941 |
|  |  | SC | -3.75167^*^ | .54776 | .000 | -4.8943 | -2.6091 |
|  |  | SP | -8.19500^*^ | .54776 | .000 | -9.3376 | -7.0524 |
|  | PP | PC | 1.73667^*^ | .54776 | .005 | .5941 | 2.8793 |
|  |  | SC | -2.01500^*^ | .54776 | .001 | -3.1576 | -.8724 |
|  |  | SP | -6.45833^*^ | .54776 | .000 | -7.6009 | -5.3157 |
|  | SC | PC | 3.75167^*^ | .54776 | .000 | 2.6091 | 4.8943 |
|  |  | PP | 2.01500^*^ | .54776 | .001 | .8724 | 3.1576 |
|  |  | SP | -4.44333^*^ | .54776 | .000 | -5.5859 | -3.3007 |
|  | SP | PC | 8.19500^*^ | .54776 | .000 | 7.0524 | 9.3376 |
|  |  | PP | 6.45833^*^ | .54776 | .000 | 5.3157 | 7.6009 |
|  |  | SC | 4.44333^*^ | .54776 | .000 | 3.3007 | 5.5859 |
| *. The mean difference is significant at the 0.05 level. | | | | | | | |

**Univariate Analysis of Variance**

| **Between-Subjects Factors** | | | |
| --- | --- | --- | --- |
|  | | Value Label | N |
| POLISH | 1 | P | 12 |
|  | 2 | S | 12 |
| SANDBLAST | 1 | C | 12 |
|  | 2 | P | 12 |

| **Tests of Between-Subjects Effects** | | | | | |
| --- | --- | --- | --- | --- | --- |
| Dependent Variable: SBS | | | | | |
| Source | Type III Sum of Squares | df | Mean Square | F | Sig. |
| Corrected Model | 224.644^a^ | 3 | 74.881 | 83.190 | .000 |
| Intercept | 6855.316 | 1 | 6855.316 | 7615.932 | .000 |
| POLISH | 156.366 | 1 | 156.366 | 173.715 | .000 |
| SANDBLAST | 57.289 | 1 | 57.289 | 63.645 | .000 |
| POLISH * SANDBLAST | 10.989 | 1 | 10.989 | 12.208 | .002 |
| Error | 18.003 | 20 | .900 |  |  |
| Total | 7097.962 | 24 |  |  |  |
| Corrected Total | 242.646 | 23 |  |  |  |
| a. R Squared = .926 (Adjusted R Squared = .915) | | | | | |
