# Supplementary figures and images for: The effect of subpressure on the bond strength of resin to zirconia ceramic
Source: PLoS One. 2017 Jun 22;12(6):e0179668. doi: 10.1371/journal.pone.0179668 (PMC5480956; doi:10.1371/journal.pone.0179668)

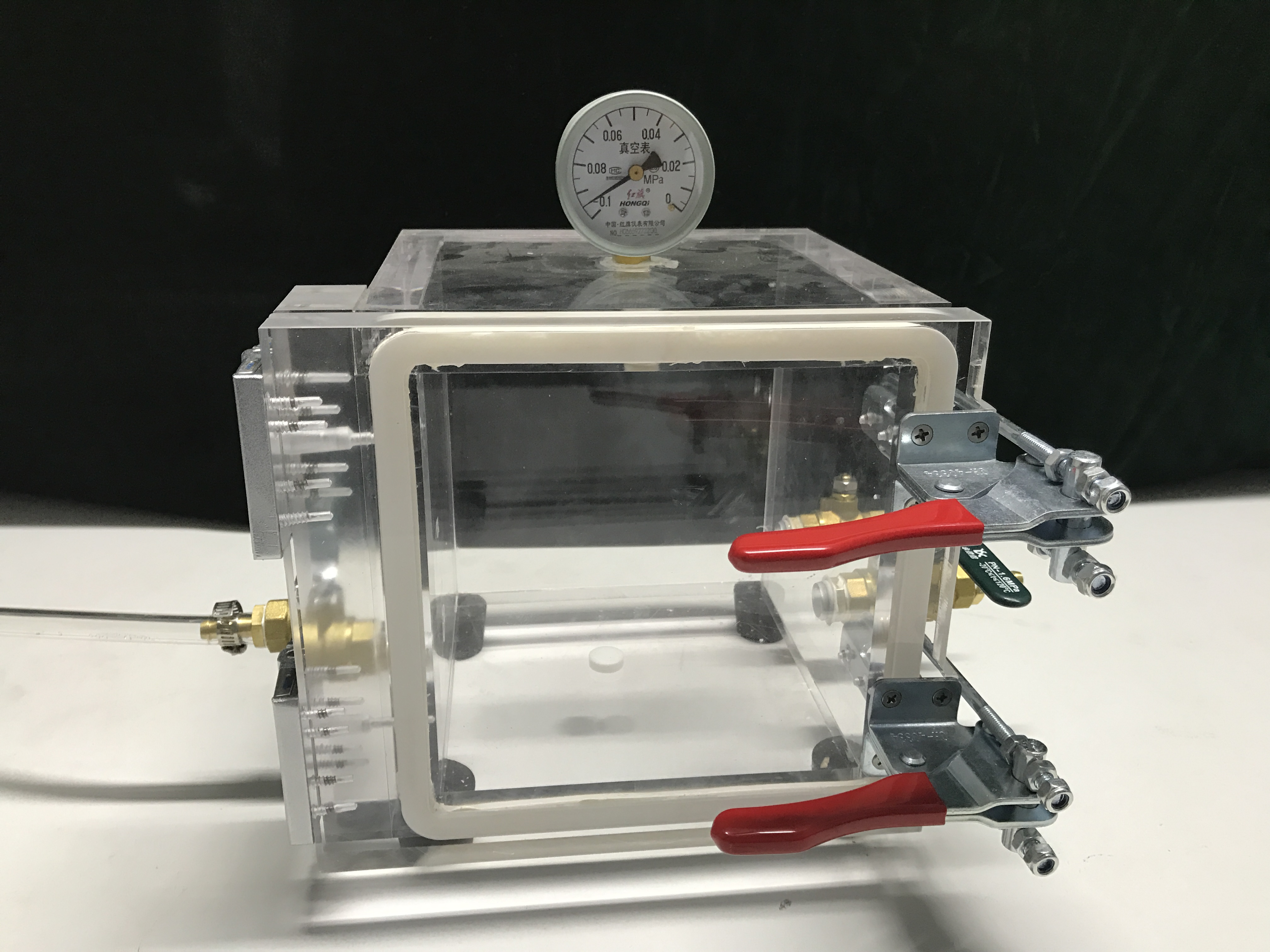

Supplement: S1 Fig — (TIF) [file pone.0179668.s002.tif]
